# Alantolactone Enhances the Phagocytic Properties of Human Macrophages and Modulates Their Proinflammatory Functions

**DOI:** 10.3389/fphar.2020.01339

**Published:** 2020-09-03

**Authors:** Barbara Gierlikowska, Wojciech Gierlikowski, Urszula Demkow

**Affiliations:** ^1^ Department of Laboratory Diagnostics and Clinical Immunology of Developmental Age, Medical University of Warsaw, Warsaw, Poland; ^2^ Department of Internal Medicine and Endocrinology, Medical University of Warsaw, Warsaw, Poland

**Keywords:** alantolactone, *Staphylococcus aureus*, THP-1, phagocytosis, intracellular killing, cytokines

## Abstract

**Aim of the Study:**

Phagocytosis is a crucial element of innate immune defense involved in bacterial killing. The aim of our study was to evaluate the influence of alantolactone on phagocytosis and cytokines release by THP1-derived macrophages. We assessed whether antimicrobial compound alantolactone (a sesquiterpene lactone present in *Inula helenium L*.) is able to stimulate immune functions of macrophages by increase of *S. aureus* uptake, phagosome acidification and further stimulation of phago-lysosomes formation. Simultaneously, we tested influence of alantolactone on cytokines/chemokines production and p65 NF-κB concentration. The activity of alantolactone was compared with clarithromycin at concentration 20 µM.

**Methods:**

The cytotoxicity of alantolactone as well as *S. aureus* uptake, pH of phagosomes and phago-lysosomes fusion were analysed with flow cytometry. Reactive oxygen species and superoxide production were evaluated spectrophotometrically. The efficiency of phagocytosis was evaluated *via* quantifying viable bacteria (CFU). The effect on p65 protein concentration and cytokine production by macrophages were measured by enzyme-linked immunosorbent assay (ELISA).

**Results:**

Alantolactone enhanced phagocytosis *via* increase of *S. aureus* uptake, acidification of phagosomes, and later stimulation of phago-lysosomes fusion. Alantolactone treatment resulted in ROS and superoxide production decrease. Furthermore, alantolactone inhibited production of pro-inflammatory cytokines TNF-α, IL-1β, IL-6, and IL-8 as well as decreased p65 concentration, the subunit responsible for NF-κB activation and cytokine production and simultaneously stimulated release of anti-inflammatory mediators (IL-10 and TGF-β).

**Conclusion:**

Results of our study indicate that alantolactone enhances clearance of *S. aureus*, and simultaneously modulates immune response, preventing collateral damage of the surrounding tissues. Considering the importance of phagocytosis in the pathogen killing, alantolactone may have a great potential as the supportive treatment of *S. aureus* infections. Further *in vivo* studies are warranted to confirm this hypothesis.

## Introduction

*Staphylococcus aureus*, one of the world’s most prolific pathogen, afflicts humans and animals’ morbidity and mortality world-wide. As previously recognized, this bacteria commonly causes nosocomial infections, but some strains have a propensity to disseminate between otherwise healthy individuals, giving rise to community-acquired illnesses (Mediavilla et al., 2012). The most serious concern occurred with the emergence of multi-drug resistant strains, such as methicillin-resistant *S. aureus* (MRSA) demonstrating enhanced infectivity and virulence (Nimmo, 2012; Francis and Kuyyalil, 2018). Incredibly, these bacteria can colonize every tissue, causing pathologies varying from whole range of skin and soft tissue infections to fatal invasive diseases, such as necrotizing pneumonia and sepsis (Gonzalez et al., 2005; Labandeira-Rey et al., 2007; Saavedra-Lozano et al., 2008). The success of *S. aureus* as a pathogen can be attributed, among others, to the ability to weaken both the innate and adaptive immune responses of the host.

Phagocytic cells, mainly neutrophils and macrophages, are essential for effective host immune response to infections. The interaction of neutrophils with *S. aureus* has been thoroughly characterized (Spaan et al., 2013). Remarkably, *S. aureus* can withstand neutrophil-mediated killing, which is an impressive feature, considering the potent microbicidal capacity of the neutrophils. In contrast, the interaction of *S. aureus* with macrophages has not been studied thoroughly. Macrophages are professional phagocytes that possess large armamentarium of antimicrobial functions, and thus represent an important component of the innate immune response. What is more, macrophages can shape adaptive immunity through phagocytosis of pathogens and presentation of their antigens (Flannagan et al., 2015). Given the immune functions of the macrophages, it stands to a reason that evasion of macrophage-dependent killing of pathogens is essential for successful initiation and maintenance of an infection.

Phagocytosis is an example of canonical defense against pathogens (Tauber, 2003). Activation of membrane toll-like receptors (TLRs) by components of bacterial cell wall, such as lipoteichoic acid, leads to rearrangement of actin cytoskeleton of macrophages, and subsequently, to the internalization of a particle in the newly-formed phagosomes (Flannagan et al., 2012). Phagosome formation is not microbicidal *per se*, as the lumen of the nascent vacuole reflects the fluid phase outside the macrophage and the surrounding phagosomal membrane is derived directly from the cell membrane. However, the nascent phagosome rapidly undergoes significant biochemical remodeling, achieved by acquisition and removal of proteins and a marked decrease in pH (Pitt et al., 1992). This process of phagosome “maturation” is comprised of a series of strictly coordinated membrane fission/fusion events between the phagosome and endo/lysosomes, and leads to the formation of the mature phagolysosome, a degradative organelle possessing potent microbicidal properties (Fairn and Grinstein, 2012). The newly created phagolysosomes may be among the main effectors of pathogens killing. This statement is based on extensive correlative data associating phago-lysosomes formation with the efficiency of the pathogen killing (Sturgill-Koszycki et al., 1994; Clemens and Horwitz, 1995; Ullrich et al., 1999; Aberdein et al., 2013).

Killing pathogens in the phagolysosomes depends on activation of the phagocyte NADPH oxidase (Nox2). The NADPH oxidase catalyzes formation of highly unstable super oxide anion (O_2_^−^) that initiates a variety of chemical reactions, resulting in the generation of noxious reactive oxygen species (ROS) such as superoxide, peroxides, hydroxyl radical, α-oxygen and singlet oxygen (Pospíšil et al., 2019). ROS production in phagocytes serves multiple purposes, from cell signaling to microbial killing. The balance between intensity and timing of ROS production versus ROS scavenging appears to be critical for the pathogen clearance (Dupré-Crochet et al., 2013).

Intracellular killing is a highly sophisticated process, which requires a complex network of molecular pathways for successful pathogen damage (Aderem, 2003). Binding of pathogen to surface receptors, especially TLR2 and TLR4, initiates a rapid pro-inflammatory response mediated by pro-inflammatory transcription factors, mainly NF-κB (Schorey and Cooper, 2003). The effects typical for early events of the immune response mediated by NF-κB include release of both, pro- and anti-inflammatory cytokines (Hayden et al., 2006; Sur et al., 2019). It is clear that some cytokines, like IL-1β, IFNs, or IL-10, modulate phagocytic properties of macrophages (Zhai et al., 2019). They may intensify the maturation of phagosomes and intracellular killing inside of phagolysosomes (Zhai et al., 2019). However, it is poorly understood how *S. aureus* modulates the production of the other cytokines, such as IL-1β, IL-6, IL-8, INF-α, IL-12, IL-10, and TGF-β.

Stimulation of phagocytic properties of macrophages may be a promising treatment strategy. According to Hanckock et al., a novel approach involves host-directed immunomodulatory therapies, whereby natural mechanisms of the host are used to enhance the therapeutic benefit (Hancock et al., 2012). The objective is to initiate or enhance phagocytic properties of leukocytes while limiting inflammation-induced tissue injury. The latter needs proper balance between pro- and anti-inflammatory cytokines (Cicchese et al., 2018).

The aim of our study was to evaluate the biological activity of alantolactone, being the dominant compound occurring in Inula species, in context of *S. aureus* infection (Kim et al., 2017; Gierlikowska et al., 2020). We have evaluated influence of alantolactone on phagocytosis and cytokines release by macrophages. We assessed whether alantolactone, known as antimicrobial compound (Stojanović-Radić et al., 2012), is able to stimulate immune response of macrophages by increase of *S. aureus* uptake, phagosome acidification and further stimulation of phago-lysosomes formation. Simultaneously, we tested influence of alantolactone on NF-κB activity and release of cytokines/chemokines. The activity of alantolactone was compared with clarithromycin tested at 20 µM. Clarithromycin is a macrolide antibiotic, which has been shown to have the anti-microbial and immunomodulating properties (Simpson et al., 2008; Cervin et al., 2009). Some data highlight the ability of clarithromycin to stimulate phagocytic properties of macrophages (Xu et al., 1996) and neutrophils (Fietta et al., 1997).

## Materials and Methods

### Chemicals and Reagents

RPMI 1640 Medium, L-glutamine, alantolactone ≥98% (HPLC), clarithromycin ≥95% (HPLC), Lysostaphin from *Staphylococcus staphylolyticus*, 12-myristate 13-acetate (PMA), DAPI solution were bought from Sigma-Aldrich Chemie GmbH (Steinheim, Germany). Fetal calf serum (FCS) and Phosphate buffered saline (PBS) were obtained from Gibco (Grand Island, NY, USA) and penicillin–streptomycin from PAA Laboratories (Pasching, Austria). Accutase™ Cell detachment solution from BD Biosciences (San Jose, CA, USA). Cellular ROS/Superoxide Detection Assays were obtained from Abcam (Cambridge, UK). *S. aureus* 25923 was bought from ATCC (Virginia, USA). Alexa 647 carboxylic acid, Alexa Fluor 633 phalloidin and TMR- and biotin-conjugated dextran were obtained from Thermo Fisher (Massachusetts, USA). NF-κB p65 Transcription Factor Assay kit was bought from Active Motif (CA, USA). Enzyme-linked immunosorbent assays (ELISA) were obtained from BD Biosciences (San Jose, USA).

### Alantolactone Source and Preparation of Stock Solutions for Bioassays

**Figure 1:**
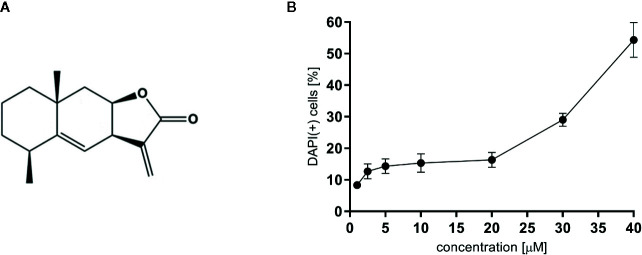
**(A)** The chemical structure of alantolactone, and **(B)** the cytotoxicity of alantolactone in THP-1 cells.

For all experiments alantolactone purchased from Sigma Aldrich was used (Steinheim, Germany). The chemical structure of alantolactone is presented in **Figure 1A**. Alantolactone was dissolved in DMSO (10 mM stock solution) and tested at a concentration range of 1–20 μM. As a positive control clarithromycin tested at 20 μM was used.

### *Staphylococcus aureus* Culture

The bacteria were cultured in basal LB medium, centrifuged and suspended in 1 mL of 0.1 M sodium bicarbonate (pH 8.3). Subsequently, 50 µL of Alexa Fluor 647 carboxylic acid (10 mg/mL DMSO) or Alexa Fluor 633 phalloidin (10 mg/mL DMSO) was added to the cell suspension and incubated for 1 h in the dark. After incubation, cells were washed 4 times in 100 mM glycine and 2 times in PBS. Eventually, bacteria were suspended in 200 µL of PBS containing 0.02% sodium azide.

### Antimicrobial Properties of Alantolactone Against *Staphylococcus aureus*

Sensitivity of *S. aureus* was examined by the standard disc-diffusion method according to CLSI (previously NCCLS) guidelines (Watts et al., 2008). The results (diameter of the growth inhibition zone) were read after 18 h of incubation at 35°C. Minimal Inhibitory Concentrations (MIC) were tested by the two fold serial microdilution method (in 96-well microtiter plates) using Mueller-Hinton Broth medium (Beckton Dickinson) according to CLSI guidelines (Watts et al., 2008). Alantolactone and clarithromycin were both tested at 20 µM.

### THP-1 Culture

THP-1, a monocytes cell line, was purchased from the ATCC collection. Cells were cultured in RPMI 1640 medium supplemented with 10% heat-inactivated fetal calf serum (FCS), penicillin and streptomycin at concentrations of 100 units/mL and 100 μg/mL respectively. Cell culturing was performed at 37°C and 5% CO_2_ in a humidified atmosphere. For differentiation, phorbol-12-myristate 13-acetate (PMA) was added to a final concentration of 10 nM. After 48 h, the PMA supplemented media were removed, cells were washed with PBS and rested in fresh PMA-free media for further 24 h in order to gain phenotypic characteristics of macrophages.

### Determination of Cytotoxicity by PI Staining

Cytotoxicity of alantolactone was determined by a standard flow cytometric measurement using DAPI staining. Differentiated THP-1 cells were seeded on 12-well plates, 5 × 10^5^ cells per well, and cultured for 24 h in a medium containing alantolactone in a concentration range 1-40 µM. After incubation, cells were harvested with Accutase, centrifuged (1300 RPM; 10 min; 4°C), labeled with DAPI and re-suspended in 400 µL of PBS. After 15 min of incubation with DAPI at room temperature, cytotoxic effect of alantolactone was analyzed by BD LSRFortessa flow cytometer (BD Biosciences, San Jose, CA, USA) by recording 10,000 events per sample. Cells that displayed high permeability to DAPI were expressed as percentage of DAPI (+) cells with the loss of cell membrane integrity.

### *S. aureus* Uptake Assay

**Figure 2:**
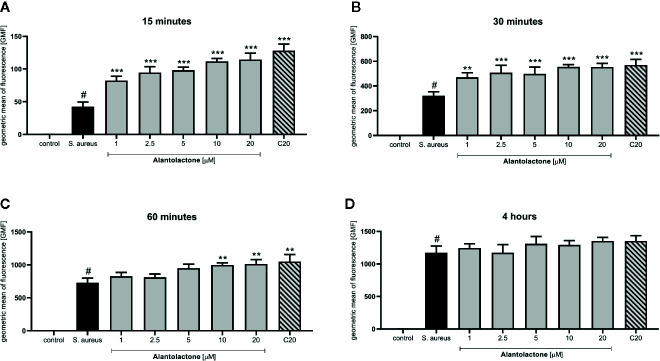
The influence of alantolactone treatment on *S. aureus* uptake by THP-1 cells. Data show geometric mean of fluorescence (GMF) phagocytosed heat-killed *S. aureus* by THP-1 cells at different time-points: **(A)** 15 min after infection, **(B)** 30 min after infection, **(C)** 60 min after infection and **(D)** 4 hours after infection. Each experimental set was compared to *S. aureus*-stimulated control.Statistical significance: *p < 0.05, **p < 0.01, ***p < 0.001 versus stimulated control, # statistically significant (p < 0.001) versus non-stimulated control (ANOVA and Dunnett’s post hoc test); control-non-stimulated control; S. aureus - *S.aureus*-stimulated control.

**Figure 3:**
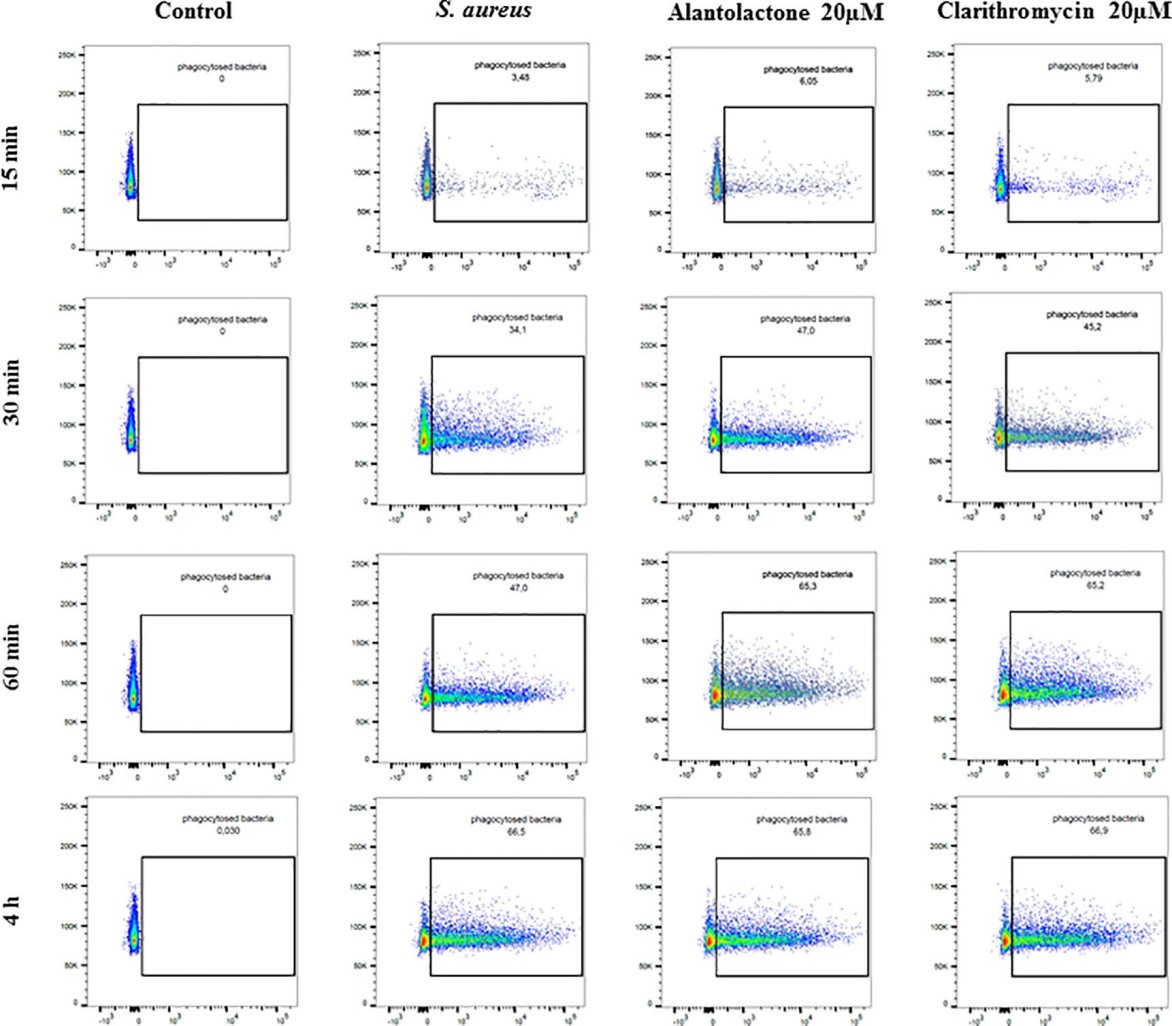
The influence of alantolactone on S. aureus uptake by THP-1 cells at different time-points (15, 30, 60 min, and 4 h). Data show representative cytograms obtained at different time-points (15, 30, 60 min, and 4 h).

Differentiated THP-1 cells were seeded on 6-well plates, 1.5 × 10^6^ cells per well. Compounds were added as described above and cells were cultured for 24 h. Subsequently, pretreated cells were infected with Alexa 647-labeled *S. aureus* for 15, 30, and 60 min and 4 h. After infection, cells were washed twice on ice with ice-cold PBS, then suspended in 400 µL of cold PBS, scraped and left in tubes on ice until flow cytometry analysis. Analysis was performed on single cells as presented in the Supplementary Material (**Figure S1**). The value of geometric mean of fluorescence (GMF) as well as representative cytograms were shown in **Figures 2** and **3**. The % of positive-infected cells (cells phagocytosed one or more bacteria) has been included in the Supplementary Material (**Figure S2**). Differences between controls (uninfected vs *S. aureus*-infected cells) were visualized by Zeiss LSM 710/780 confocal microscopy after prior cell fixation with 4% formaldehyde and DAPI staining.

### Phagosomal pH Measurements

For phagosomal pH measurement, macrophages were seeded on 12-well plates as described above, pretreated for 24 h with compounds (or calibration buffers) and then incubated for 1 h with heat-killed *S. aureus*-labeled with Alexa Fluor 647. Intracellular pH calibration was achieved by clamping phagosomal pH using potassium rich buffer solutions (120 mM KCl, 20 mM NaCl, 1 mM CaCl_2_, 1 mM MgCl_2_, 10 mM HEPES with the pH adjusted from 4 to 8) for 30 min at 37°C. Changes in pH were measured by the intensity ratios of fluorescence using flow cytometry. The method was adapted from Di et all protocol (Di et al., 2006; Renna et al., 2011).

### Phagosome-Lysosome Fusion Assay

For phagosome-lysosome fusion analysis, differentiated THP-1 cells were seeded on 12-well plates as described above, they were pretreated for 24 h with compounds and then incubated (1-h pulse, 4-h chase) with TMR- and biotin-conjugated dextran to load lysosomes and then fed IgG-coated fluorescent latex beads (30-min pulse, 2-h chase). Beads were recovered by cell disruption, the degree of bound fluorescent dextran was quantified by flow cytometry, and average geometric mean fluorescence was determined.

### Superoxide and Reactive Oxygen Species (ROS) Production

For superoxide and reactive oxygen species (ROS) measurement, differentiated THP-1 were seeded on 96-well plates at a density 2 × 10^4^ cells per well. After 24 h supernatants were removed from cells and cells were carefully washed, treated with alantolactone for 30 min and then infected with *S. aureus* for further 1 h. Generation of superoxide and total reactive oxygen species production in the real-time in live cells were quantified using a Cellular ROS/Superoxide kit. Detection Assays were performed according to the manufacturer’s instructions. Fluorescence was measured using BioTek Synergy 4 Plate Reader (CA, USA) at Ex = 488 nm, Em = 520 nm and Ex = 550 nm, Em = 610 nm for the detection of ROS and superoxide, respectively. Results were expressed as percentage of fluorescence intensity normalized to the fluorescence of the positive control (*S. aureus*-infected cells).

### *S. aureus* Killing Assay

#### No Pre-Treated Differentiated THP-1 Cells

Differentiated THP-1 cells, seeded on 12-well plates as described above, were inoculated with *S. aureus* (MOI 1:1) for 1 h at 37°C, then washed with PBS twice, treated with 10 µg/mL lysostaphin for 30 min, washed with PBS twice and then treated for 24 h at 37°C with 0.5 µg/mL lysostaphin in the presence or absence of alantolactone or clarithromycin, as indicated. We serially diluted the suspension and plated 20 μL on TYE agar plates. Bacteria were counted after incubation at 37°C for 24 h and the amount was calculated as CFU (colony forming unit).

#### Pre-Treated Differentiated THP-1 Cells

Differentiated THP-1 cells were seeded as described above, they were pre-incubated with alantolactone or clarithromycin for 24 h at 37°C, then inoculated with *S. aureus* (MOI 1:1) for 12 h at 37°C, then washed with PBS twice, treated with 10 µg/mL lysostaphin for 30 min, washed with PBS twice and then treated for 24 h at 37°C with 0.5 µg/mL lysostaphin in the presence or absence of alantolactone or clarithromycin, as indicated. We serially diluted the suspension and plated 20 μL on TYE agar plates. Bacteria were counted after incubation at 37°C for 24 h and the amount was calculated as CFU (colony forming unit).

### Pro- and Anti-Inflammatory Cytokine Production

Differentiated THP-1 cells were seeded on 12-well plates and infected with *S. aureus* (MOI 1:1). After 1 h, cells were treated with alantolactone or clarithromycin and cultured for 24 h. Subsequently, medium was collected and the amount of released cytokines was measured using the OptEIA™ Set (BD Biosciences, USA), which contains the components necessary to develop enzyme-linked immunosorbent assays (ELISA). The results were expressed as a percentage of the released protein compared to the *S. aureus*-stimulated control.

### p65 NF-κB Concentration

Differentiated THP-1 cells were seeded on 12-well plates and infected with *S. aureus* (MOI 1:1). After 1 h, cells were treated with alantolactone or clarithromycin for 24 h. Cells were washed with ice-cold PBS, suspended in hypotonic buffer (20 mM HEPES, 5 mM NaF, 10 µM Na_2_MoO_4_, 0.1 mM EDTA) and left on ice for 15 min. Next, cells were centrifuged and supernatant (cytoplasmic fraction) was removed. The nuclear pellets were suspended in complete lysis buffer supplemented with protease inhibitor cocktail and left on ice for 30 min. Pellets were centrifuged for 10 min 2500 RPM and stored at -80°C for further analysis. The protein concentration was determined by using Bradford-based assay, following manufacturer’s protocol.

10 µg of nuclear fraction was incubated in 96-well plates pre-coated with immobilized oligonucleotide containing a consensus binding site for p65, following the manufacturer’s instructions (Active Motif, CA, USA). The nuclear fractions were incubated with the primary antibody (1:1000 dilution) for 1 h, washed 3 times with wash buffer provided by manufacturer and incubated with HRP-conjugated antibody (1:1000 dilution) for 1 h. After incubation, plate was washed 3 times, reaction was stopped by adding stop solution and concentration of NF-κB p65 was measured spectrophotometrically at 450 nm.

### Statistical Analysis

The results were expressed as the mean ± SEM from three independent experiments assayed in triplicates. All analyses were performed using Statistica 13.1 software. GraphPad Prism (version 5.01) was used to plot data. The statistical significance of the differences between means was established by ANOVA with Dunnett’s *post hoc* test. P values below 0.05 were considered statistically significant.

## Results

### Alantolactone Suppresses *S. aureus* Growth

To validate the direct impact of alantolactone on *S. aureus* viability, growth inhibition zone (GIZ) was measured. The GIZ was visible for *S. aureus* treated with alantolactone and clarithromycin tested at 20 µM. GIZ was estimated to be 14 mm for alantolactone and 23 mm for clarithromycin. Minimal inhibitory concentration (MIC) was detectable only for clarithromycin and was estimated as 0.25 µg/mL (~ 0.36 µM). Obtained results were comparable with reference values present at EUCAST (The European Committee on Antimicrobial Susceptibility Testing).

### Cytotoxicity

To evaluate cytotoxic effect of alantolactone, DAPI staining was performed. Alantolactone in concentrations up to 20 μM did not influence the viability of differentiated THP-1 cells after 24 h incubation (**Figure 1B**). At 20 µM, we noticed 13.67 ± 4.93% DAPI-positive cells, which expressed % of cells with damaged cellular membrane (dead and/or necrotic) (**Figure 1**) The IC_50_ concentration against tested cells may be estimated to be 38 µM. Clarithromycin tested at 20 µM induced 10,33 ± 2,52% DAPI-positive cells.

### Alantolactone Stimulates *S. aureus* Uptake

**Figure 4:**
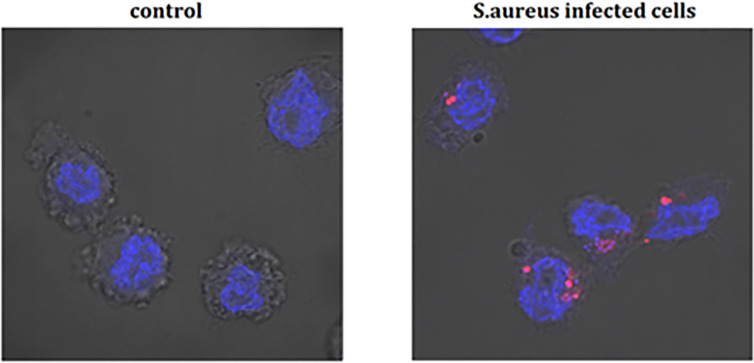
The infection of THP-1 cells by *S. aureus*. Data show photographed controls (not infected cells) and *S. aureus* infected cells (15 min after infection). The blue marker—DAPI-labelled nucleus, the pink marker—Alexa 647 labelled-*S. aureus*.

The canonical immune response of macrophages to bacterial infection is phagocytosis. The effect of alantolactone on *S. aureus* uptake was most pronounced at the beginning of the treatment (approximately 2 times higher uptake compared to *S. aureus* treated cells at 15 min) (**Figures 2**, **3**, **4** and **Figure S3**). The number of uptaken bacteria was alantolactone dose- dependent. After 1 h, the dynamics of phagocytosis changed and macrophages started to accumulate more bacteria than they were able to phagocyte (overaccumulation).

### Alantolactone Treatment Leads to Phagosome Acidification

**Figure 5:**
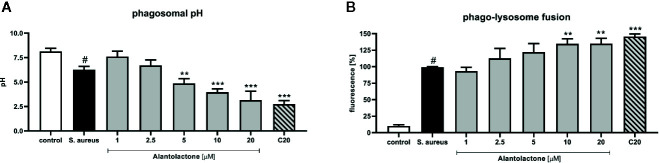
The influence of alantolactone on **(A)** phagosomal pH and **(B)** phago-lysosome fusion. Statistical significance: *p < 0.05, **p < 0.01, ***p < 0.001 versus stimulated control (ANOVA and Dunnett’s *post hoc* test); control—non-stimulated control; *S. aureus*—*S.aureus*-stimulated control. # statistically significant (p < 0.001) versus non-stimulated control.

Phagosomal pH of THP-1 cells was measured in the response to heat-killed *S. aureus* suspended in sodium azide. THP-1 cells were incubated with buffer solutions for the pH adjustment from 4 to 8 for intracellular calibration. We confirmed that treatment with alantolactone (up to 5 µM) strongly stimulates phagosomal acidification (p < 0.01) (**Figure 5A**). Similarly to uptake results, the level of acidification was dose-dependent. The pH of the highest alantolactone concentrations (10 and 20 µM) were comparable to pH of clarithromycin tested at 20 µM.

### Alantolactone Treatment Intensifies Phago-Lysosome Fusion

Matured and acidified phagosomes fuse with lysosomes forming phagolysosomes. To exclude non-specific quenching of fluorescence, analysis was performed with *S. aureus* suspended in sodium azide, as described above. *S. aureus* infection increased the phago-lysosomes fusion (**Figure 5B**). Alantolactone at the highest concentrations (10 and 20 μM) stimulated phago-lysosomal fusion by about 30%.

### Alantolactone Treatment Decreases Superoxide and Reactive Oxygen Species (ROS) Production

**Figure 6:**
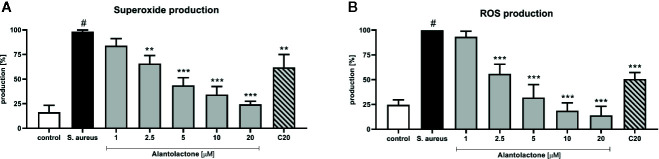
The influence of alantolactone on **(A)** superoxide and **(B)** ROS production by *S.aureus*-stimulated THP-1 cells. Statistical significance: *p < 0.05, **p < 0.01, ***p < 0.001 versus stimulated control (ANOVA and Dunnett’s *post hoc* test); control—non-stimulated control; *S. aureus*—*S.aureus*-stimulated control. # statistically significant (p < 0.001) versus non-stimulated control.

Stimulation of macrophages with *S. aureus* resulted in increased ROS production by these cells (**Figures 6A, B**). Alantolactone treatment resulted in the reduction of the oxidative stress. The drug in dose above 2.5 µM attenuated ROS production equally to clarithromycin at 20 µM. Summarizing, the suppression of ROS production by alantolactone may maintain phagocytic properties of macrophages (especially if the infection is at an early stage).

### Alantolactone Treatment Enhances Phagocytosis Efficiency

**Figure 7:**
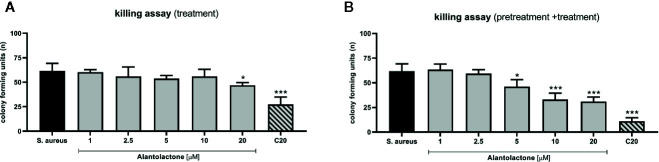
The effect of alantolactone on *S. aureus* phagocytosis efficiency performed by **(A)** THP-1 cells treated with alantolactone after infection **(B)** and THP-1 cells pretreated before infection and treated after infection. Statistical significance: *p < 0.05, **p < 0.01, ***p < 0.001 versus stimulated control (ANOVA and Dunnett’s *post hoc* test); *S. aureus*—*S. aureus*-stimulated control.

The evaluation of phagocytosis efficiency was performed on THP-1 cells infected with *S. aureus*, as described above. The measurement of CFU value was performed 24 h after starting treatment (**Figures 7A, B**). Obtained results clearly indicate that alantolactone significantly decreased CFU value at 12 h after the treatment onset, and the effect was maintained at the later time points (18 and 24 h after treatment). Clarithromycin significantly decreased CFU value, and biological effect was detectable earlier than for alantolactone (6 h after starting treatment).

### Alantolactone Modulates Cytokine Production and p65 NF-κB Concentration

**Figure 8:**
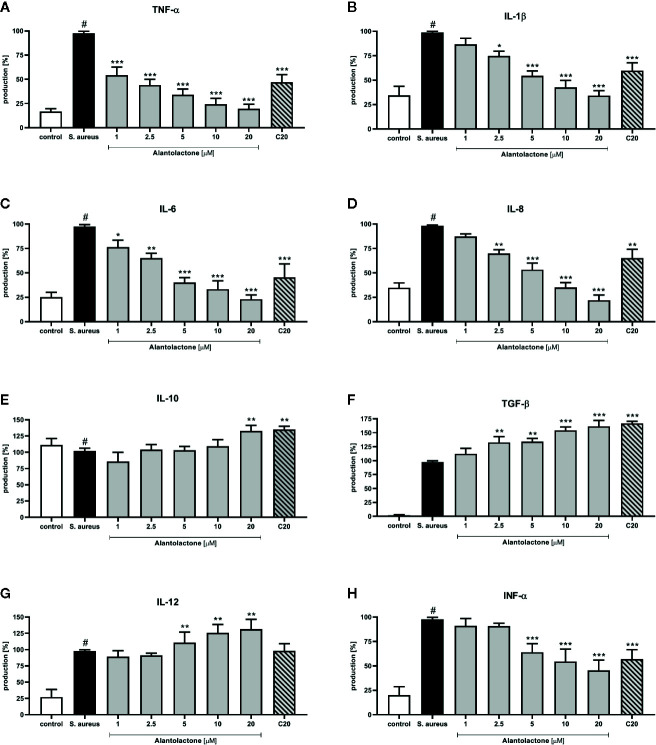
The influence of alantolactone on the pro- and anti-inflammatory cytokines production by THP-1 cells **(A**–**H)** Statistical significance: *p < 0.05, **p < 0.01, ***p < 0.001 versus stimulated control (ANOVA and Dunnett’s *post hoc* test); control—non-stimulated control; *S. aureus*—*S. aureus*-stimulated control. # statistically significant (p < 0.001) versus non-stimulated control.

The stimulation with *S. aureus* resulted in a statistically significant induction of cytokines and chemokines release. Alantolactone treatment resulted in a suppression of selected pro-inflammatory cytokines (TNF-α, IL-1β, IL-6, IL-8, and INF-α) production (**Figure 8**). For concentrations above 5 µM, production of mentioned cytokines was suppressed by alantolactone in dose-dependent manner. At 1 µM (the lowest tested concentration) alantolactone was able to suppress TNF-α and IL-6 with significance p < 0.001 for TNF-α and p < 0.05 for IL-6, respectively (**Figures 8A, C**). Importantly, our observations indicated that alantolactone tested at the concentration > 5 µM stimulated IL-12 production (**Figure 8G**). Alantolactone also stimulated the production of anti-inflammatory cytokines IL-10, but only at the highest concentration (20 µM); moreover, it increased TGF-β release at the concentration > 2.5 µM (**Figures 8E, F**).

Clarithromycin at the concentration of 20 μM suppressed TNF-α, IL-1β, IL-6, and INF-α production (p < 0.001) and IL-8 production (p < 0.01) (in comparison to stimulated control 100% of release). Additionally, clarithromycin enhanced production of IL-10 and TGF-β (**Figures 8E, F**), and observed effect was comparable to alantolactone tested at 20 µM. These observations indicate the potential anti-inflammatory properties of clarithromycin (mainly known as drug with strong anti-microbial properties).

**Figure 9:**
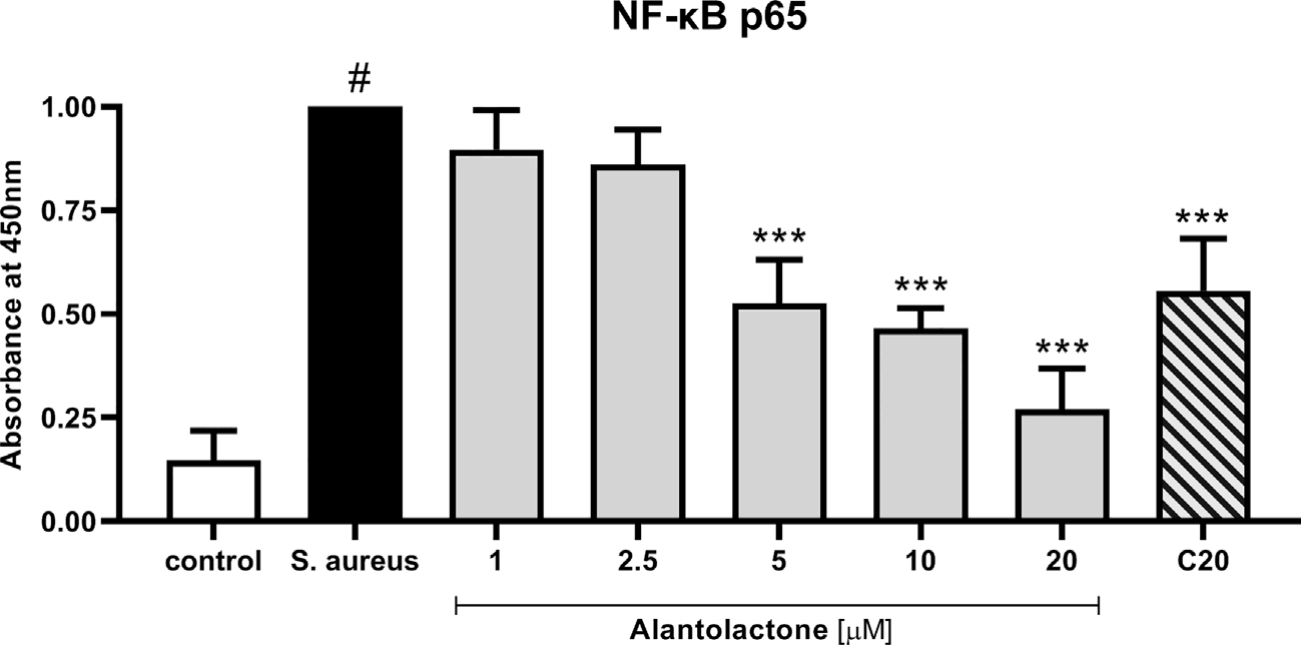
The influence of alantolactone on p65 NF-κB concentration after *S. aureus* infection. Statistical significance: *p < 0.05, **p < 0.01, ***p < 0.001 versus stimulated control (ANOVA and Dunnett’s *post hoc* test); control—non-stimulated control; *S. aureus*—*S. aureus*-stimulated control. # statistically significant (p < 0.001) versus non-stimulated control.

Evaluation of p65 NF-κB concentration explains the molecular mechanism responsible for previously observed cytokine concentration changes. Our data show that alantolactone at the concentration above 5 µM significantly decreased p65 production (p < 0.001) (**Figure 9**). Similarly, clarithromycin tested at 20 µM significantly inhibited p65 production, and final effect was comparable to alantolactone tested at 5 µM.

**Figure 10:**
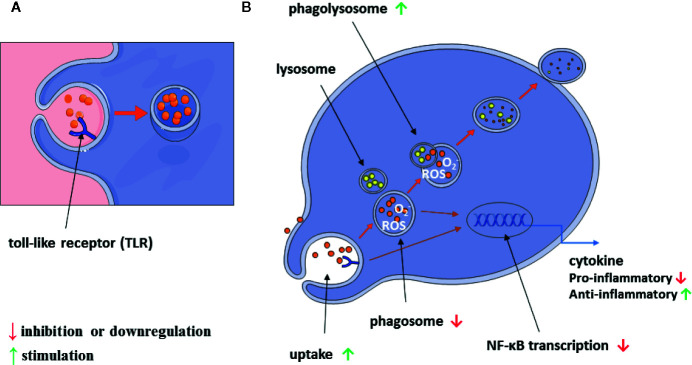
Scheme of alantolactone influence on macrophage immunomodulatory and inflammatory properties **(A, B)**.

A graphical summary of our results is shown in **Figure 10**.

## Discussion

The immunomodulatory characteristics of plant-based therapeutics have gathered the attention of researchers. Innovative technologies and the excessive research on immunomodulatory natural products, plants, their extracts, and their active moieties with immunomodulatory potential, have provided valuable entities to develop novel immunomodulatory agents to supplement the present therapies. The immunomodulatory properties of phagocytes can be enhanced pharmacologically (Xu et al., 1996).

Many plant-derived medications (plant extracts, compounds) show immunomodulatory, anti-inflammatory and anti-microbial properties, thus can be considered as promising and novel anti-*S. aureus* compounds. An example of plant with documented anti-*S. aureus* activity is *Inula helenium L.* (Asteraceae) (O’Shea et al., 2009). Root of *I. helenium* was used in the traditional medicine to treat bacteria-induced respiratory tract infections (Gierlikowska et al., 2020). *I. helenium* extract relieves symptoms of bronchial and throat infection, bronchitis, catarrh and colds (Ivancheva and Stantcheva, 2000; Leporatti and Ivancheva, 2003; Jarić et al., 2015). It also has an antitussive effect and aids coughing up of mucus (Seca et al., 2014; Shikov et al., 2014; Pranskuniene et al., 2018).

The phytochemical analysis clearly indicated alantolactone as a dominant compound in the plant material (alantolactone, 52.4%) (Bourrel et al., 1993). According to the records of the European and China Pharmacopoeia (Wang et al., 2017), alantolactone possesses a wide range of biological properties, such as anti-inflammatory, antibacterial, antifungal and immunomodulatory activities. Chemically, alantolactone belongs to sesquiterpene lactones including the α-methylene-γ-lactone group, which may potentially predispose to delayed hypersensitivity (Dupuis et al., 1980; Warshaw and Zug, 1996), but also exerts strong anti-inflammatory effects by interaction with NF-κB transcription factor (Siedle et al., 2004). Chun et al. reported the anti-inflammatory properties of alantolactone and proved that alantolactone at 10 µM suppresses inducible nitric oxide and cyclooxygenase 2 (COX-2) expression by down-regulating mitogen-activated protein kinase (MAPK), NF-κB and activator protein 1 (AP-1) in LPS-stimulated RAW 264.7 cells (Chun et al., 2012). Lim et al. (2015) reported that alantolacone at 5 µM down-regulates STAT1 signaling in TNF-α and IFN-γ-stimulated cells. The STATs regulate various aspects of growth, survival, and differentiation in cells (Dai et al., 2006). Some studies demonstrated that STAT proteins are involved in the development and function of the immune system and play a role in maintaining immune tolerance (Dai et al., 2008). Chun et al. (2015) highlighted the ability of alantolactone to inhibit STAT3, which makes this compound a potential therapeutic agent against breast cancer.

Although alantolactone has well-documented anti-inflammatory properties, it was unknown how it regulates immunomodulatory functions of phagocytes. To answer this question, we used a standard THP-1 cell line, infected with *S. aureus* and then treated with alantolactone. Our main findings explain how alantolactone influences molecular mechanisms involved in phagocytosis.

We confirmed that at low micromolar concentrations (1–20 µM) alantolactone intensifies uptake of *S. aureus*. The effect was the most pronounced especially at the beginning of the observation (approximately 2 times higher uptake compared to *S. aureus* treated cells at 15 min). The prolonged infection time (over 1 h) may lead to overaccumulation of *S. aureus* inside of macrophages and impairment of phagocytic properties of macrophages. The literature confirms the paralysis of the phagocytosis in the prolonged infection. According to Jubrail et al. (2016) at low MOI (closely related to the beginning of infection) macrophages can kill almost all ingested *S. aureus*, but the capacity to up-regulate killing is decreased as the bacterial load grows. Moreover, some intracellular bacteria may develop the potential to survive inside macrophages (Ibarra and Steele-Mortimer, 2009; Pumerantz et al., 2011). Thus, the pharmacological enhancement of uptake of *S. aureus* at the early time point (15 min) may significantly accelerate redirecting pathogens to phagolysosomes to remove them successfully.

Additionally, we observed that alantolactone treatment leads to acidification of phagosomes. In macrophages, in which phagosome acidification is impaired, the response to *S. aureus* can be significantly limited (Ip et al., 2010). Taken together, these observations delineate the inter-dependence of phagocytosis with pH of phagosomes and suggest that therapeutics augmenting functions and biochemical properties of phagosomes may be useful in increasing host response to *S. aureus*.

It is worth emphasizing that the phagosomal pH changes in response to both dead (heat-killed, as we tested) (Renna et al., 2011) and alive bacteria (Jubrail et al., 2016). However, for scientific purposes heat-killed are recommended. Accurate measurement of phagosomal pH is challenging, mainly due to the myeloperoxidase (MPO) production by phagocytes (Sugiyama et al., 2001). The respiratory burst produces H_2_O_2_, which reacts with MPO and chloride anions form hypochlorous acid (Takeshita et al., 2006). The chlorination of compounds results in a quenching of fluorescence as well as a shift in their spectral properties, giving a false indication of acidification (Hurst et al., 1984). This unwanted effect can be avoided by inhibiting MPO directly with sodium azide (Nauseef et al., 1983), as we did (see Materials and Methods: *Staphylococcus aureus* culture).

Our next finding was that alantolactone stimulates phagolysosome formation/fusion. The phagolysosome formation is crucial for further intracellular pathogen killing and successful clearance (Jordao et al., 2008; Clarke and Weiser, 2011). Thus, pharmacological stimulation of this process (by alantolactone or other compound/s) may potentially enhance the phagocytosis efficiency. The obtained finding is strengthened by the knowledge, that *S.aureus* possesses diverse mechanisms, allowing avoidance of destruction in phagolysosomes (Staali et al., 2006).

Although alantolactone inhibits ROS production inside the phagosomes and phagolysosomes, it does not decrease the final phagocytosis efficiency. ROS functions are clearly concentration-dependent (Radak et al., 2013). Low amounts of ROS are essential for activation of molecular pathways responsible for intracellular microbial killing (Dupré-Crochet et al., 2013). However, the excessive amounts of ROS lead to oxidative stress and damage of phagocytic functions of immune cells, as well as surrounding host’s tissues. The antioxidants of immune cells play a pivotal role in the protection against oxidative stress and therefore preserving their adequate functions. Thus, decrease of ROS amounts during bacterial infection may potentially protect phagocytes, with their phagocytic functions, and other host’s tissues from dysfunction (Chakraborty et al., 2012).

Signal transduction by ROS often takes place on a subcellular scale over periods of seconds or minutes, thus in our experimental variant (1 h after infection) decrease of ROS is consistent with previous observations (Wu et al., 2012) and prognoses the appropriate function of ROS. Thus, attenuation of over-production of oxygen species may increase phagocytosis and in consequence decrease bacterial viability.

When macrophages are exposed to bacterial infection, they secrete a wide spectrum of pro- and anti-inflammatory cytokines. Cytokines are multi-functional signaling molecules, which play a crucial role during infection and inflammation progression. Cytokines modulate phagocytic functions of immune cells, thus the appropriate cytokine concentration may enhance pathogen eradication and decrease morbidity and/or mortality (Hübel et al., 2002). Recent data indicate that the low amounts of TNF-α and IL-1β increase phagocytes chemotaxis, enable intracellular phagosome maturation and stimulate phagolysosome formation, necessary for final intracellular killing (Kaufmann and Dorhoi, 2016). Also, TNF-α, IL-1β, IL-6, IL-8 may activate oxidative and non-oxidative metabolic responses of immune cells to pathogens (Seider et al., 2011). Wahl et al. documented enhancement of phagolysosome degradative properties by INFs and TGF-β (Wahl et al., 2004). It is known that cytokines promote adequate immune response, but prolonged cytokines production by leukocytes and damaged tissues may exacerbate inflammation, leading to cytokine storm. The side effects arise from excessive leukocyte chemotaxis and over-stimulation of their pro-inflammatory and immunomodulatory functions.

Our findings indicate that alantolactone significantly modulates cytokines production *via* p65 NF-κB suppression. Based on our experiments performed on a simplified *in vitro* model, we can speculate that alantolactone at early phase of infection may potentially decrease chemotaxis of leukocytes, but, on the other hand, phagocytes are very sensitive to cytokines released to bloodstream, thus even small amounts of secreted cytokines may activate adequate immune response. Stimulation of IL-10 and TGF-β during development of inflammation is beneficial for homeostasis, as both cytokines stimulate macrophages migration and clearance of apoptotic neutrophils, pathogens and damaged tissues, contributing to the effective counterbalance of inflammation.

Comparing the activity of alantolactone with clarithromycin, it is worth to emphasize the promising competitiveness of phytotherapy. The searching of a new biological properties of plant-derived compounds has been experiencing a renaissance in recent years (Bocanegra-García et al., 2009). The differences between phagocytosis modulated by clarithromycin- and alantolactone-treated cells can be explained by the fact, that clarithromycin can be accumulated in phagocytic cells and possesses significant intracellular bactericidal activity for a long time. In contrast to clarithromycin, alantolactone is poorly absorbed by the cells (Zhou et al., 2018). Thus, it is more probable, that the observed effect for alantolactone is a consequence of modulation of the phagocytic functions of the cells, not of the direct effect of compound on bacteria.

Sesquiterpene lactones are potent NF-κB inhibitors, which determines their anti-inflammatory properties, but so far, they are not classified as immunomodulators. Till now, there was no data regarding modulation of phagocytosis by sesquiterpene lactones. Among known plant-derived compounds, some were identified as potential immunomodulators, including flavonoids, alkaloids, diterpenoids, polysaccharides and glycosides. Examples of plant-derived compounds, which have exhibited potent effects on cellular and humoral immune functions in pre-clinical investigations, are curcumin, resveratrol, quercetin, colchicine, capsaicin, andrographolide, genistein and artemisinin (Jantan et al., 2015).

To conclude, it becomes obvious that alantolactone exerts immunomodulatory and anti-inflammatory effect *via* multiple pathways. Alantolactone enhances phagocytosis, one of the main components of innate immune response, and simultaneously modulates p65 NF-κB activation affecting pro- and anti-inflammatory cytokine production involved in phagolysosome formation. We postulate that such additive pharmacodynamic effects can be beneficial for the patients with the *S. aureus* infections.

## Data Availability Statement

The raw data supporting the conclusions of this article will be made available by the authors, without undue reservation, to any qualified researcher.

## Author Contributions

BG conceived the study and obtained financial support. BG and WG performed the biological experiments, evaluated data and drafted the manuscript. UD critically revised the manuscript. All authors contributed to the article and approved the submitted version.

## Funding

This research was partially financed by The European Union’s Erasmus+ programme and Willmar Schwabe Research Scholarship for Young Scientists 2018 by the Society for Medicinal Plant and Natural Product Research (GA).

## Conflict of Interest

The authors declare that the research was conducted in the absence of any commercial or financial relationships that could be construed as a potential conflict of interest.

## Abbreviations

ROS, Reactive Oxygen Spices; SO, Superoxide; CFU, colony forming unit; MOI, multiplicity of infection; NF-κB, nuclear factor kappa-light-chain-enhancer of activated B cells; TNFα, tumor necrosis alpha; IL-6, interleukin 6; IL-8, interleukin 8; IL-1β, interleukin 1β; IL-10, interleukin 10; IL-12, interleukin 12; TGF-β, transforming growth factor beta, INF-α, interferon alfa; PMA, Phorbol 12-myristate 13-acetate; DMSO, dimethyl sulfoxide; FCS, fetal calf serum; PBS, phosphate buffered saline.

## References

[B1] Aberdein J. Cole J. Bewley M. Marriott H. Dockrell D. (2013). Alveolar macrophages in pulmonary host defence–the unrecognized role of apoptosis as a mechanism of intracellular bacterial killing. Clin. Exp. Immunol. 174 (2), 193–202. 23841514 10.1111/cei.12170 PMC3828822

[B2] Aderem A. (2003). Phagocytosis and the inflammatory response. J. Infect. Dis. 187 (Suppl 2), S340–S345. 10.1086/374747 12792849

[B3] Bocanegra-García V. Del Rayo Camacho-Corona M. Ramírez-Cabrera M. Rivera G. Garza-González E. (2009). The bioactivity of plant extracts against representative bacterial pathogens of the lower respiratory tract. BMC Res. Notes 2, 95. 10.1186/1756-0500-2-95 19486533 PMC2702266

[B4] Bourrel C. Vilarem G. Perineau F. (1993). Chemical analysis, bacteriostatic and fungistatic properties of the essential oil of elecampane (Inula helenium L.). J. Essent. Oil Res. 5 (4), 411–417.

[B5] Cervin A. Wallwork B. Mackay-Sim A. Coman W. B. Greiff L. (2009). Effects of long-term clarithromycin treatment on lavage-fluid markers of inflammation in chronic rhinosinusitis. Clin. Physiol. Funct. Imaging 29 (2), 136–142. 10.1111/j.1475-097X.2008.00848.x 19076731

[B6] Chakraborty S. P. Mahapatra S. K. Roy S. (2012). In vitro time-dependent vancomycin-resistant Staphylococcus aureus-induced free radical generation and status of antioxidant enzymes in murine peritoneal macrophage. Toxicol. Mech. Methods 22 (1), 9–22. 10.3109/15376516.2011.583296 21958328

[B7] Chun J. Choi R. J. Khan S. Lee D.-S. Kim Y.-C. Nam Y.-J. (2012). Alantolactone suppresses inducible nitric oxide synthase and cyclooxygenase-2 expression by down-regulating NF-κB, MAPK and AP-1 via the MyD88 signaling pathway in LPS-activated RAW 264.7 cells. Int. Immunopharmacol. 14 (4), 375–383. 10.1016/j.intimp.2012.08.011 22940184

[B8] Chun J. Li R.-J. Cheng M.-S. Kim Y. S. (2015). Alantolactone selectively suppresses STAT3 activation and exhibits potent anticancer activity in MDA-MB-231 cells. Cancer Lett. 357 (1), 393–403. 10.1016/j.canlet.2014.11.049 25434800

[B9] Cicchese J. M. Evans S. Hult C. Joslyn L. R. Wessler T. Millar J. A. (2018). Dynamic balance of pro- and anti-inflammatory signals controls disease and limits pathology. Immunol. Rev. 285 (1), 147–167. 10.1111/imr.12671 30129209 PMC6292442

[B10] Clarke T. B. Weiser J. N. (2011). Intracellular sensors of extracellular bacteria. Immunol. Rev. 243 (1), 9–25. 10.1111/j.1600-065X.2011.01039.x 21884164

[B11] Clemens D. L. Horwitz M. A. (1995). Characterization of the Mycobacterium tuberculosis phagosome and evidence that phagosomal maturation is inhibited. J. Exp. Med. 181 (1), 257–270. 10.1084/jem.181.1.257 7807006 PMC2191842

[B12] Dai X. Sayama K. Yamasaki K. Tohyama M. Shirakata Y. Hanakawa Y. (2006). SOCS1-negative feedback of STAT1 activation is a key pathway in the dsRNA-induced innate immune response of human keratinocytes. J. Invest. Dermatol. 126 (7), 1574–1581. 10.1038/sj.jid.5700294 16628196

[B13] Dai X. Sayama K. Tohyama M. Shirakata Y. Yang L. Hirakawa S. (2008). The NF-kappaB, p38 MAPK and STAT1 pathways differentially regulate the dsRNA-mediated innate immune responses of epidermal keratinocytes. Int. Immunol. 20 (7), 901–909. 10.1093/intimm/dxn048 18492658

[B14] Di A. Brown M. E. Deriy L. V. Li C. Szeto F. L. Chen Y. (2006). CFTR regulates phagosome acidification in macrophages and alters bactericidal activity. Nat. Cell Biol. 8 (9), 933–944. 10.1038/ncb1456 16921366

[B15] Dupré-Crochet S. Erard M. Nüβe O. (2013). ROS production in phagocytes: why, when, and where? J. Leukoc. Biol. 94 (4), 657–670. 10.1189/jlb.1012544 23610146

[B16] Dupuis G. Benezra C. Schlewer G. Stampf J. L. (1980). Allergic contact dermatitis to alpha-methylene-gamma-butyrolactones. Preparation of alantolactone-protein conjugates and induction of contact sensitivity in the guinea pig by an alantolactone-skin protein conjugate. Mol. Immunol. 17 (8), 1045–1051. 10.1016/0161-5890(80)90099-1 6108504

[B17] Fairn G. D. Grinstein S. (2012). How nascent phagosomes mature to become phagolysosomes. Trends Immunol. 33 (8), 397–405. 10.1016/j.it.2012.03.003 22560866

[B18] Fietta A. Merlini C. Gialdroni Grassi G. (1997). Inhibition of intracellular growth of Staphylococcus aureus by exposure of infected human monocytes to clarithromycin and azithromycin. J. Chemother. 9 (1), 17–22. 10.1179/joc.1997.9.1.17 9106013

[B19] Flannagan R. S. Jaumouillé V. Grinstein S. (2012). The cell biology of phagocytosis. Annu. Rev. Pathol. 7, 61–98. 10.1146/annurev-pathol-011811-132445 21910624

[B20] Flannagan R. S. Heit B. Heinrichs D. E. (2015). Antimicrobial Mechanisms of Macrophages and the Immune Evasion Strategies of Staphylococcus aureus. Pathog. (Basel Switzerland) 4 (4), 826–868. 10.3390/pathogens4040826 PMC4693167 26633519

[B21] Francis D. Kuyyalil S. (2018). Immunogenicity and protective efficacy of recombinant alkaline shock protein 23 from Staphylococcus aureus in a murine model. Cent. Eur. J. Immunol. 43 (4), 371–377. 10.5114/ceji.2018.81348 30799984 PMC6384426

[B22] Gierlikowska B. Gierlikowski W. Bekier K. Skalicka-Woźniak K. Czerwińska M. E. Kiss A. K. (2020). Inula helenium and Grindelia squarrosa as a source of compounds with anti-inflammatory activity in human neutrophils and cultured human respiratory epithelium. J. Ethnopharmacol. 249, 112311. 10.1016/j.jep.2019.112311 31644941

[B23] Gonzalez B. E. Martinez-Aguilar G. Hulten K. G. Hammerman W. A. Coss-Bu J. Avalos-Mishaan A. (2005). Severe Staphylococcal sepsis in adolescents in the era of community-acquired methicillin-resistant Staphylococcus aureus. Pediatrics 115 (3), 642–648. 10.1542/peds.2004-2300 15741366

[B24] Hancock R. E. Nijnik A. Philpott D. J. (2012). Modulating immunity as a therapy for bacterial infections. Nat. Rev. Microbiol. 10 (4), 243–254. 10.1038/nrmicro2745 22421877

[B25] Hayden M. S. West A. P. Ghosh S. (2006). NF-kappaB and the immune response. Oncogene 25 (51), 6758–6780. 10.1038/sj.onc.1209943 17072327

[B26] Hübel K. Dale D. C. Liles W. C. (2002). Therapeutic use of cytokines to modulate phagocyte function for the treatment of infectious diseases: current status of granulocyte colony-stimulating factor, granulocyte-macrophage colony-stimulating factor, macrophage colony-stimulating factor, and interferon-gamma. J. Infect. Dis. 185 (10), 1490–1501. 10.1086/340221 11992286

[B27] Hurst J. K. Albrich J. M. Green T. R. Rosen H. Klebanoff S. (1984). Myeloperoxidase-dependent fluorescein chlorination by stimulated neutrophils. J. Biol. Chem. 259 (8), 4812–4821. 6325409

[B28] Ibarra J. A. Steele-Mortimer O. (2009). Salmonella–the ultimate insider. Salmonella virulence factors that modulate intracellular survival. Cell Microbiol. 11 (11), 1579–1586. 10.1111/j.1462-5822.2009.01368.x 19775254 PMC2774479

[B29] Ip W. K. Sokolovska A. Charriere G. M. Boyer L. Dejardin S. Cappillino M. P. (2010). Phagocytosis and phagosome acidification are required for pathogen processing and MyD88-dependent responses to Staphylococcus aureus. J. Immunol. 184 (12), 7071–7081. 10.4049/jimmunol.1000110 20483752 PMC2935932

[B30] Ivancheva S. Stantcheva B. (2000). Ethnobotanical inventory of medicinal plants in Bulgaria. J. Ethnopharmacol. 69 (2), 165–172. 10.1016/s0378-8741(99)00129-4 10687872

[B31] Jantan I. Ahmad W. Bukhari S. N. (2015). Plant-derived immunomodulators: an insight on their preclinical evaluation and clinical trials. Front. Plant Sci. 6, 655. 10.3389/fpls.2015.00655 26379683 PMC4548092

[B32] Jarić S. Mačukanović-Jocić M. Djurdjević L. Mitrović M. Kostić O. Karadžić B. (2015). An ethnobotanical survey of traditionally used plants on Suva planina mountain (south-eastern Serbia). J. Ethnopharmacol. 175, 93–108. 10.1016/j.jep.2015.09.002 26375774

[B33] Jordao L. Bleck C. K. Mayorga L. Griffiths G. Anes E. (2008). On the killing of mycobacteria by macrophages. Cell Microbiol. 10 (2), 529–548. 10.1111/j.1462-5822.2007.01067.x 17986264

[B34] Jubrail J. Morris P. Bewley M. A. Stoneham S. Johnston S. A. Foster S. J. (2016). Inability to sustain intraphagolysosomal killing of Staphylococcus aureus predisposes to bacterial persistence in macrophages. Cell Microbiol. 18 (1), 80–96. 10.1111/cmi.12485 26248337 PMC4778410

[B35] Kaufmann S. H. E. Dorhoi A. (2016). Molecular Determinants in Phagocyte-Bacteria Interactions. Immunity 44 (3), 476–491. 10.1016/j.immuni.2016.02.014 26982355

[B36] Kim M. Song K. Kim Y. S. (2017). Alantolactone Improves Prolonged Exposure of Interleukin-6-Induced Skeletal Muscle Inflammation Associated Glucose Intolerance and Insulin Resistance. Front. Pharmacol. 8, 405. 10.3389/fphar.2017.00405 28706484 PMC5489625

[B37] Labandeira-Rey M. Couzon F. Boisset S. Brown E. L. Bes M. Benito Y. (2007). Staphylococcus aureus Panton-Valentine leukocidin causes necrotizing pneumonia. Science 315 (5815), 1130–1133. 10.1126/science.1137165 17234914

[B38] Leporatti M. L. Ivancheva S. (2003). Preliminary comparative analysis of medicinal plants used in the traditional medicine of Bulgaria and Italy. J. Ethnopharmacol. 87 (2-3), 123–142. 10.1016/s0378-8741(03)00047-3 12860298

[B39] Lim H. S. Jin S. E. Kim O. S. Shin H. K. Jeong S. J. (2015). Alantolactone from Saussurea lappa Exerts Antiinflammatory Effects by Inhibiting Chemokine Production and STAT1 Phosphorylation in TNF-α and IFN-γ-induced in HaCaT cells. Phytother. Res. 29 (7), 1088–1096. 10.1002/ptr.5354 25881570

[B40] Mediavilla J. R. Chen L. Mathema B. Kreiswirth B. N. (2012). Global epidemiology of community-associated methicillin resistant Staphylococcus aureus (CA-MRSA). Curr. Opin. Microbiol. 15 (5), 588–595. 10.1016/j.mib.2012.08.003 23044073

[B41] Nauseef W. M. Metcalf J. A. Root R. K. (1983). Role of myeloperoxidase in the respiratory burst of human neutrophils. Blood 61 (3), 483–492. 6297637

[B42] Nimmo G. R. (2012). USA300 abroad: global spread of a virulent strain of community-associated methicillin-resistant Staphylococcus aureus. Clin. Microbiol. Infect. 18 (8), 725–734. 10.1111/j.1469-0691.2012.03822.x 22448902

[B43] O’Shea S. Lucey B. Cotter L. (2009). In vitro activity of Inula helenium against clinical Staphylococcus aureus strains including MRSA. Br. J. BioMed. Sci. 66 (4), 186–189. 10.1080/09674845.2009.11730271 20095126

[B44] Pitt A. Mayorga L. S. Stahl P. D. Schwartz A. L. (1992). Alterations in the protein composition of maturing phagosomes. J. Clin. Invest. 90 (5), 1978–1983. 10.1172/jci116077 1430221 PMC443261

[B45] Pospíšil P. Prasad A. Rác M. (2019). Mechanism of the Formation of Electronically Excited Species by Oxidative Metabolic Processes: Role of Reactive Oxygen Species. Biomolecules 9 (7), 258. 10.3390/biom9070258 PMC6681336 31284470

[B46] Pranskuniene Z. Dauliute R. Pranskunas A. Bernatoniene J. (2018). Ethnopharmaceutical knowledge in Samogitia region of Lithuania: where old traditions overlap with modern medicine. J. Ethnobiol. Ethnomed. 14 (1), 70. 10.1186/s13002-018-0268-x 30458833 PMC6247776

[B47] Pumerantz A. Muppidi K. Agnihotri S. Guerra C. Venketaraman V. Wang J. (2011). Preparation of liposomal vancomycin and intracellular killing of meticillin-resistant Staphylococcus aureus (MRSA). Int. J. Antimicrob. Agents 37 (2), 140–144. 10.1016/j.ijantimicag.2010.10.011 21130608

[B48] Radak Z. Zhao Z. Koltai E. Ohno H. Atalay M. (2013). Oxygen consumption and usage during physical exercise: the balance between oxidative stress and ROS-dependent adaptive signaling. Antioxid. Redox Signal 18 (10), 1208–1246. 10.1089/ars.2011.4498 22978553 PMC3579386

[B49] Renna M. Schaffner C. Brown K. Shang S. Tamayo M. H. Hegyi K. (2011). Azithromycin blocks autophagy and may predispose cystic fibrosis patients to mycobacterial infection. J. Clin. Invest. 121 (9), 3554–3563. 10.1172/jci46095 21804191 PMC3163956

[B50] Saavedra-Lozano J. Mejías A. Ahmad N. Peromingo E. Ardura M. I. Guillen S. (2008). Changing trends in acute osteomyelitis in children: impact of methicillin-resistant Staphylococcus aureus infections. J. Pediatr. Orthop. 28 (5), 569–575. 10.1097/BPO.0b013e31817bb816 18580375

[B51] Schorey J. S. Cooper A. M. (2003). Macrophage signalling upon mycobacterial infection: the MAP kinases lead the way. Cell Microbiol. 5 (3), 133–142. 10.1046/j.1462-5822.2003.00263.x 12614457

[B52] Seca A. M. Grigore A. Pinto D. C. Silva A. M. (2014). The genus Inula and their metabolites: from ethnopharmacological to medicinal uses. J. Ethnopharmacol. 154 (2), 286–310. 10.1016/j.jep.2014.04.010 24754913

[B53] Seider K. Brunke S. Schild L. Jablonowski N. Wilson D. Majer O. (2011). The facultative intracellular pathogen Candida glabrata subverts macrophage cytokine production and phagolysosome maturation. J. Immunol. 187 (6), 3072–3086. 10.4049/jimmunol.1003730 21849684

[B54] Shikov A. N. Pozharitskaya O. N. Makarov V. G. Wagner H. Verpoorte R. Heinrich M. (2014). Medicinal plants of the Russian Pharmacopoeia; their history and applications. J. Ethnopharmacol. 154 (3), 481–536. 10.1016/j.jep.2014.04.007 24742754

[B55] Siedle B. García-Piñeres A. J. Murillo R. Schulte-Mönting J. Castro V. Rüngeler P. (2004). Quantitative structure-activity relationship of sesquiterpene lactones as inhibitors of the transcription factor NF-kappaB. J. Med. Chem. 47 (24), 6042–6054. 10.1021/jm049937r 15537359

[B56] Simpson J. L. Powell H. Boyle M. J. Scott R. J. Gibson P. G. (2008). Clarithromycin targets neutrophilic airway inflammation in refractory asthma. Am. J. Respir. Crit. Care Med. 177 (2), 148–155. 10.1164/rccm.200707-1134OC 17947611

[B57] Spaan A. N. Surewaard B. G. Nijland R. van Strijp J. A. (2013). Neutrophils versus Staphylococcus aureus: a biological tug of war. Annu. Rev. Microbiol. 67, 629–650. 10.1146/annurev-micro-092412-155746 23834243

[B58] Staali L. Bauer S. Mörgelin M. Björck L. Tapper H. (2006). Streptococcus pyogenes bacteria modulate membrane traffic in human neutrophils and selectively inhibit azurophilic granule fusion with phagosomes. Cell Microbiol. 8 (4), 690–703. 10.1111/j.1462-5822.2005.00662.x 16548894

[B59] Stojanović-Radić Z. Comić L. Radulović N. Blagojević P. Denić M. Miltojević A. (2012). Antistaphylococcal activity of Inula helenium L. root essential oil: eudesmane sesquiterpene lactones induce cell membrane damage. Eur. J. Clin. Microbiol. Infect. Dis. 31 (6), 1015–1025. 10.1007/s10096-011-1400-1 21901633

[B60] Sturgill-Koszycki S. Schlesinger P. H. Chakraborty P. Haddix P. L. Collins H. L. Fok A. K. (1994). Lack of acidification in Mycobacterium phagosomes produced by exclusion of the vesicular proton-ATPase. Science 263 (5147), 678–681. 10.1126/science.8303277 8303277

[B61] Sugiyama S. Okada Y. Sukhova G. K. Virmani R. Heinecke J. W. Libby P. (2001). Macrophage myeloperoxidase regulation by granulocyte macrophage colony-stimulating factor in human atherosclerosis and implications in acute coronary syndromes. Am. J. Pathol. 158 (3), 879–891. 10.1016/s0002-9440(10)64036-9 11238037 PMC1850342

[B62] Sur L. M. Sur G. Samasca G. Lupan I. (2019). Investigations of cellular immunity in juvenile idiopathic arthritis. Cent. Eur. J. Immunol. 44 (1), 92–96. 10.5114/ceji.2019.83615 31114442 PMC6526591

[B63] Takeshita J. Byun J. Nhan T. Q. Pritchard D. K. Pennathur S. Schwartz S. M. (2006). Myeloperoxidase generates 5-chlorouracil in human atherosclerotic tissue: a potential pathway for somatic mutagenesis by macrophages. J. Biol. Chem. 281 (6), 3096–3104. 10.1074/jbc.M509236200 16326702

[B64] Tauber A. I. (2003). Metchnikoff and the phagocytosis theory. Nat. Rev. Mol. Cell Biol. 4 (11), 897–901. 10.1038/nrm1244 14625539

[B65] Ullrich H. J. Beatty W. L. Russell D. G. (1999). Direct delivery of procathepsin D to phagosomes: implications for phagosome biogenesis and parasitism by Mycobacterium. Eur. J. Cell Biol. 78 (10), 739–748. 10.1016/s0171-9335(99)80042-9 10569246

[B66] Wahl S. M. Swisher J. McCartney-Francis N. Chen W. (2004). TGF-beta: the perpetrator of immune suppression by regulatory T cells and suicidal T cells. J. Leukoc. Biol. 76 (1), 15–24. 10.1189/jlb.1103539 14966194

[B67] Wang X. Yu Z. Wang C. Cheng W. Tian X. Huo X. (2017). Alantolactone, a natural sesquiterpene lactone, has potent antitumor activity against glioblastoma by targeting IKKβ kinase activity and interrupting NF-κB/COX-2-mediated signaling cascades. J. Exp. Clin. Cancer Res. 36 (1), 93. 10.1186/s13046-017-0563-8 28701209 PMC5508758

[B68] Warshaw E. M. Zug K. A. (1996). Sesquiterpene lactone allergy. Am. J. Contact Dermat. 7 (1), 1–23. 10.1016/s1046-199x(96)90028-7 8796737

[B69] Watts J. Shryock T. Apley M. Bade D. Brown S. Gray J. (2008). Performance standards for antimicrobial disk and dilution susceptibility tests for bacteria isolated from animals: approved standard. (Clinical Lab Standards Institute).

[B70] Wu Y. Zhai H. Wang Y. Li L. Wu J. Wang F. (2012). Aspirin-triggered lipoxin A₄ attenuates lipopolysaccharide-induced intracellular ROS in BV2 microglia cells by inhibiting the function of NADPH oxidase. Neurochem. Res. 37 (8), 1690–1696. 10.1007/s11064-012-0776-3 22552474

[B71] Xu G. Fujita J. Negayama K. Yuube K. Hojo S. Yamaji Y. (1996). Effect of macrolide antibiotics on macrophage functions. Microbiol. Immunol. 40 (7), 473–479. 10.1111/j.1348-0421.1996.tb01097.x 8865152

[B72] Zhai W. Wu F. Zhang Y. Fu Y. Liu Z. (2019). The Immune Escape Mechanisms of Mycobacterium Tuberculosis. Int. J. Mol. Sci. 20 (2), 1–18. 10.3390/ijms20020340 PMC6359177 30650615

[B73] Zhou B. Ye J. Yang N. Chen L. Zhuo Z. Mao L. (2018). Metabolism and pharmacokinetics of alantolactone and isoalantolactone in rats: Thiol conjugation as a potential metabolic pathway. J. Chromatogr. B. Analyt. Technol. BioMed. Life Sci. 1072, 370–378. 10.1016/j.jchromb.2017.11.039 29223921

