# Supplementary material for: Alantolactone Enhances the Phagocytic Properties of Human Macrophages and Modulates Their Proinflammatory Functions
Source: Front Pharmacol. 2020 Sep 3;11:1339. doi: 10.3389/fphar.2020.01339 (PMC7494907; doi:10.3389/fphar.2020.01339)
Supplement: Supplementary file 1 [file DataSheet_1.docx]

**SUPLEMMENTARY MATERIAL**

**Alantolactone enhance the phagocytic properties of human macrophages and modulate their proinflammatory functions**

Barbara Gierlikowska^1*^, Wojciech Gierlikowski^2^ , Urszula Demkow^1^

^1^Department of Laboratory Diagnostics and Clinical Immunology of Developmental Age, Medical University of Warsaw, Banacha 1, 02-097 Warsaw, Poland,

^2^Department of Internal Medicine and Endocrinology, Medical University of Warsaw, Banacha 1a, 02-097 Warsaw, Poland

**Figure S1.** All experiments were performed on the single cells as gated below.


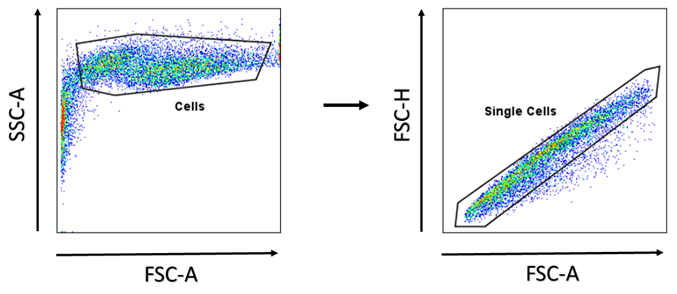


**Figure S2.** The uptake of S. aureus by THP-1-derived macrophages. The data show % of positive-infected macrophages (cells which phagocytosed one or more bacteria).

**
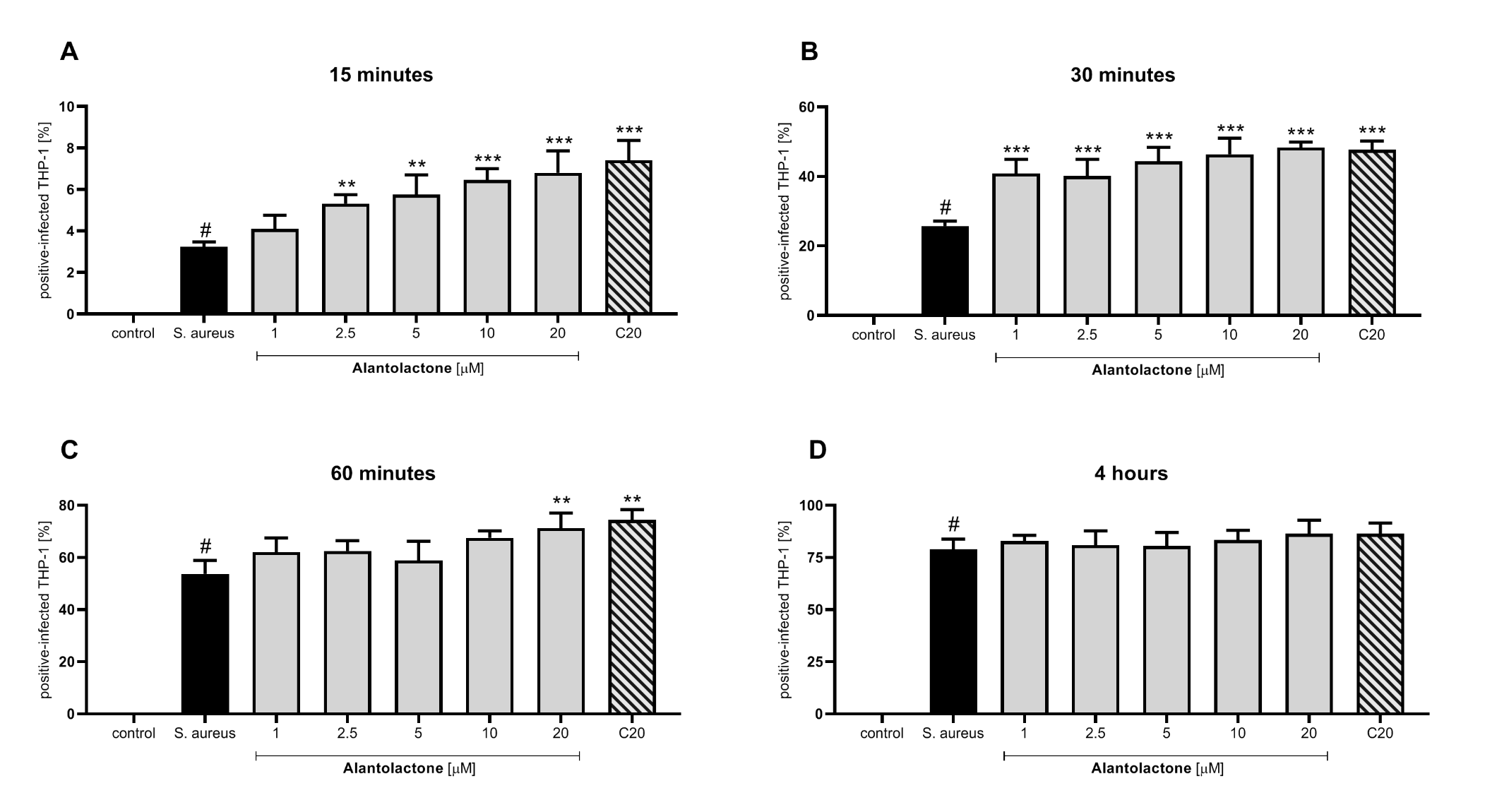
**
